# Factors associated with costs and health outcomes in patients with Back and leg pain in primary care: a prospective cohort analysis

**DOI:** 10.1186/s12913-019-4257-0

**Published:** 2019-06-21

**Authors:** Jesse Kigozi, Kika Konstantinou, Reuben Ogollah, Kate Dunn, Lewis Martyn, Susan Jowett

**Affiliations:** 1 Health Economics Unit, Institute of Applied Health Research, University of Birmingham, Edgbaston, Birmingham, B15 2TT UK; 2 Arthritis Research UK Primary Care Centre, Research Institute of Primary Care and Health Sciences, Keele University, Keele, Staffordshire UK; 3 Keele Clinical Trials Unit, David Weatherall Building, Keele University, Keele, Staffordshire ST5 5BG UK; 4 Nottingham Clinical Trials Unit, School of Medicine, University of Nottingham, Nottingham, NG7 2UH UK

**Keywords:** Cost, Outcome, Back pain, Leg pain, And sciatica

## Abstract

**Background:**

There is limited research on the economic burden of low back-related leg pain, including sciatica. The aim of this study was to describe healthcare resource utilisation and factors associated with cost and health outcomes in primary care patients consulting with symptoms of low back-related leg pain including sciatica.

**Methods:**

This study is a prospective cohort of 609 adults visiting their family doctor with low back-related leg pain, with or without sciatica in a United Kingdom (UK) Setting. Participants completed questionnaires, underwent clinical assessments, received an MRI scan, and were followed-up for 12-months. The economic analysis outcome was the quality-adjusted life year (QALY) calculated from the EQ-5D-3 L data obtained at baseline, 4 and 12-months. Costs were measured based on patient self-reported information on resource use due to back-related leg pain and results are presented from a UK National Health Service (NHS) and Societal perspective. Factors associated with costs and outcomes were obtained using a generalised linear model.

**Results:**

Base-case results showed improved health outcomes over 12-months for the whole cohort and slightly higher QALYs for patients in the sciatica group. NHS resource use was highest for physiotherapy and GP visits, and work-related productivity loss highest from a societal perspective. The sciatica group was associated with significantly higher work-related productivity costs. Cost was significantly associated with factors such as self-rated general health and care received as part of the study, while quality of life was significantly predicted by self-rated general health, and pain intensity, depression, and disability scores.

**Conclusions:**

Our results contribute to understanding the economics of low back- related leg pain and sciatica and may provide guidance for future actions on cost reduction and health care improvement strategies.

**Trial registration:**

13/09/2011 Retrospectively registered; ISRCTN62880786.

**Electronic supplementary material:**

The online version of this article (10.1186/s12913-019-4257-0) contains supplementary material, which is available to authorized users.

## Background

Low back pain (LBP) is a common health condition worldwide [1] and poses a significant burden on individuals’ quality of life [2]. In primary care, 60% of patients presenting with LBP also report leg pain, a proportion of those patients will have sciatic pain [3]. The presence of both, low back-related leg pain and sciatica is linked with increased pain, disability, poorer health outcomes, and work absence, compared to LBP without symptoms in the leg(s) [4], and consequently contributes significantly to the economic burden on individuals and society in general. Patients with low back-related leg pain not attributed to sciatica are labelled as having ‘referred’ (non-specific) leg pain, we will use this term when we describe this group.

The economic burden of LBP has been investigated comprehensively, and found to be substantial [5, 6]. To date, there is limited research on the economic burden of low back-related leg pain, including sciatica. A previous Dutch study estimated societal sciatica costs of about 13% of overall LBP related costs, equivalent to a current annual impact on the United Kingdom (UK) economy of over £500 million in healthcare costs and £3.8 billion in indirect costs related to sciatica [7, 8]. Given the socioeconomic impact of sciatica, a number of trials have investigated the effectiveness of available treatments, from medications to surgery [9–11], and national and international guidelines recommend timely assessment of patients presenting with sciatica in primary care [12, 13]. There is however limited research on the determinants of quality of life and cost outcomes in LBP patients, and specifically few studies have investigated patients presenting with sciatica or with referred leg pain, in the primary care setting. A prospective description of costs and quality of life outcomes of this patient group will improve understanding of healthcare determinants, societal costs and health outcomes, and inform health care policy and planning.

The objective of this study is to provide data on the distribution of healthcare usage, costs and quality of life outcomes in primary care patients consulting with low back-related leg pain according to the presence/absence of sciatica, and explores factors associated with costs and quality of life outcomes for patients with symptoms of low back-related leg pain including sciatica.

## Methods

### Cohort study design and population

Full details of this prospective cohort study are published in the ATLAS (**A**ssessment and **T**reatment of **L**eg pain **A**ssociated with the **S**pine) study protocol [14], and results papers [15, 16]. The study’s aims and methods are briefly described here. Ethical Approval for this study was obtained from the South Birmingham Research Ethics Committee (REC ref. 10/H1207/82). The primary objective of the ATLAS study was to investigate overall prognosis of patients seeking care for symptoms of low back-related leg pain, including sciatica. The study population consisted of 609 adults aged 18 years and over with symptoms of low back-related leg pain, including sciatica, of any severity and duration, recruited from the National Health Service (NHS) general practices in North Staffordshire and Stoke-on-Trent. Patient recruitment was carried out between April 2011 and March 2013. Adults, visiting their General Practitioner (GP) with low back-related leg pain including sciatica, of any duration and intensity, who met pre-set study criteria, were invited to participate. At a research clinic, all study patients after giving written informed consent were assessed by a physiotherapist to confirm eligibility [15, 16]. Patients also received a magnetic resonance imaging (MRI) scan (within 10 working days) as part of the research study, providing there were no contraindications to the procedure. Patients were clinically diagnosed as having sciatica or referred (non-specific) leg pain based on the examiner’s clinical opinion. Participation in the study did not infer any particular treatment advantage for the participants, treatments and treatment pathways were based on best clinical practice. In terms of care pathways, most patients received a course of physiotherapy, a small number of patients were referred to a specialist spinal service for consideration of spinal injections and/or surgery, and a small number of patients proceeded to have these procedures.

### Overview of economic analysis

An economic regression-based analysis of costs and quality of life outcomes was conducted alongside the observational cohort study. The economic analysis used costs and QALYs as the main outcomes, with the analysis conducted from an NHS and societal perspective and designed to capture data at 4 and 12 months. The main focus of the analysis was to investigate the distribution of resource use and quality of life outcomes in these patients with referred leg pain or sciatica, and the predictors of costs and quality of life outcomes in the whole cohort, with a secondary analysis focusing on the subgroup clinically diagnosed with sciatica.

### Quality of life outcome data

Preference-based health outcome data were collected at baseline, 4 and 12 months follow-up, using the patient completed EQ-5D-3 L questionnaire. The EQ-5D-3 L is a generic instrument measuring and valuing health related quality of life [17]. Responses from individuals were converted to utility values obtained using the UK value set derived from a UK general population survey [18] and expressed in QALYs using the area-under-the-curve approach linking utility scores at various time-points [19]. Adjustment for differences between patients with or without sciatica in baseline EQ-5D-3 L scores was performed using a regression-based adjustment in order to avoid bias [20]. Other secondary outcomes were also collected using self-completed questionnaires [21–27] (see Additional file 1).

### Resource use and cost data

Resource-use data due to low back-related leg pain/sciatica were collected from participants at 4 and 12 months from the time of recruitment into the study, using self-report postal questionnaires. The questionnaires specifically requested information on low back-related leg pain/sciatica healthcare resource utilisation including primary care consultations (e.g. GP, practice nurse, physiotherapist), prescribed medication, over-the-counter treatments and secondary care attendances including healthcare professionals (e.g. hospital consultants and physiotherapists), investigations (e.g. e.g. X-rays, MRI scans), and procedures such as surgery (injections, surgeries). Self-reported work-related data on time-off work were also collected in order to assess the impact of indirect costs of sickness absence due to low back related leg pain. Details of the number of study-related physiotherapy sessions attended by each participant were collected as part of the study through case report forms and costed separately from other physiotherapy visits. Study protocol-driven MRI scan costs were excluded from the analysis as these would not necessarily occur in usual practice.

Total 12-month costs per person were estimated by combining resource use data with unit costs. Unit costs were obtained from the British National Formulary (BNF) [28] for drugs, and the NHS Reference costs [29] and Unit Costs of Health and Social Care [30] for other resource use items (see Additional file 2). Productivity costs were estimated using the human capital approach (wage cost per day multiplied by the number of absence days), salary costs were based on respondent job-specific wage estimates identified from annual earnings data and UK Standard Occupational Classification coding [31–33]. Out-of-pocket treatment costs were based on patient reported costs. All costs were expressed in 2013/2014 UK (£) prices.

### Data analysis

An analysis of costs and quality of life outcomes was conducted to determine the difference in costs and QALYs over a 12-month period between the group of patients with sciatica and those with referred leg pain. Multiple-imputation using chained equations was used to impute missing values for costs and EQ-5D-3 L scores for non-responders to the 4 and 12-month questionnaires [34]. Confidence intervals for the mean differences in resource use and costs were obtained by bias corrected and accelerated non-parametric bootstrapping, using 1000 replications [35]. Discounting was not performed because of the 12 month follow-up period. Descriptive statistics (mean, standard deviation) and 95% confidence intervals for resource use, costs and QALYs are presented for the whole cohort and separately for the sciatica and referred leg pain groups.

Separate GLMs with log link and gamma variance functions were fitted for the whole cohort, to identify factors that influence total costs and QALYs [36]. GLM models account for non-normality in the outcome variables. Factors to be examined were selected a-priori based on evidence of their association with costs and health-related quality of life outcomes, building on evidence from previous studies, and expertise within the study team (Additional file 1). GLMs were used to examine the relationship of each factor with the cost (NHS and societal) and QALYs. Factors with *p* < 0.25 were carried forward to the multivariable model for each cost and quality of life outcome variables. The final models reported significance at *p* < 0.01, *p* < 0.05 and *p* < 0.1. All statistical analyses were performed using Stata V.13 analysis software [37]. The base-case analysis was from a UK NHS perspective, using the imputed cost and QALY dataset while adjusting for baseline EQ-5D-3 L scores and a secondary analysis from a societal perspective. As part of sensitivity analysis, separate models were fitted to identify predictive cost and outcome factors for the sciatica group only.

## Results

A total of 609 participants ((sciatica (*n* = 452), referred leg pain (*n* = 157)) formed the dataset for the analysis. The mean age of participants was 50, with over half of the respondent’s female (62%); 32% were smokers and just over 13% had two or more other health problems. At baseline, 61 and 59% of respondents were in paid employment in the sciatica and referred leg pain group. An additional file describes the participant’s characteristics at baseline in more detail (see Additional file 3). All base-case analyses reflect the imputed dataset unless otherwise stated.

### Health-related quality of life outcomes

Mean EQ-5D-3 L scores at baseline and follow-up time-points and mean QALYs are shown in Table 1. The distribution of quality of life outcomes was similar for the overall cohort and the sciatica and referred leg pain groups. Health-related quality of life improved over time in the whole cohort and in the two groups. The unadjusted and imputed mean QALYs over 12 months were marginally higher among the referred leg pain compared to the sciatica group. This result changed in favour of the sciatica group after adjusting for baseline differences.

**Table 1:** Mean (SD) health-related quality of life outcomes per patient over 12 months

EQ-5D-3 L scores	ALL	Referred leg pain	Sciatica
Base case (Imputed) analysis	*n* = 609	*n* = 157	*s* = 452
Baseline	0.446 (0.316)	0.470 (0.306)	0.438 (0.320)
4 months	0.625 (0.266)	0.644 (0.237)	0.613 (0.275)
12 months	0.663 (0.243)	0.649 (0.232)	0.669 (0.246)
Total 12 month QALYs			
Unadjusted	0.608 (0.228)	0.617 (0.214)	0.606 (0.232)
Difference (Bootstrapped 95% CI’s)		−0.011 (− 0.049 to 0.031)	
Adjusted^a^	0.608	0.605	0.610
Difference (Bootstrapped 95% CI’s)		0.005 (−0.021 to 0.032)	

^a^The values are predicted mean scores obtained from the multiple regression equation when controlling for baseline imbalances

*QALY* quality-adjusted life years, *CI* confidence intervals

### Resource use and costs

Table 2 shows the disaggregated details of mean resource use for participants with complete resource use data. Overall, the whole cohort analysis showed that health care resource use was highest for GP consultations, outpatient consultations, medication and physiotherapy visits not related to the study. Similar findings were observed in the two groups, with the sciatica group being associated with more GP visits, investigations (MRIs and X-rays not related to the study protocol) and prescribed medications (Table 2). Over the 12-month period, the mean (SD) total NHS cost for all patients in the cohort was £296.56 (326.75) (Table 3). NHS costs were highest for non-study related physiotherapy visits, outpatient consultation, GP consultations and investigations.

**Table 2:** Health care resource use over 12 months (mean, SD)

Resource use					
Variable	ALL	Referred leg pain		Sciatica	
	*n* = 368	*n* %	*n* = 85	*n* %	*n* = 283
ATLAS physiotherapy^a^	3.38 (2.34)		2.80 (2.08)		3.56 (2.39)
NHS					
GP consultations	2.12 (3.17)	42 (49)	1.48 (2.59)	161 (56)	2.31 (3.29)
Nurse consultations	0.14 (0.60)	10 (12)	0.22 (0.81)	18 (7)	0.11 (0.53)
Other primary care consultations	0.81 (2.36)	15 (18)	0.93 (2.95)	52 (19)	0.77 (2.16)
Outpatient consultations^b^	3.15 (4.78)	40 (47)	2.73 (4.26)	160 (56)	3.28 (4.93)
‘Other’ physiotherapy^d^	2.48 (3.90)	35 (42)	2.13 (3.35)	130 (46)	2.59 (4.06)
Consultations other^b^	0.07 (0.35)	3 (7)	0.07 (0.26)	6 (4)	0.07 (0.37)
Private					
Outpatient consultations^b^	1.18 (4.96)	8 (10)	1.82 (7.62)	40 (15)	0.99 (3.82)
‘Other’ Physiotherapy^d^ consultations	0.44 (2.19)	4 (5)	0.47 (2.77)	21 (7)	0.43 (1.99)
Consultations other^b^	0.05 (0.41)	0	0.00	5 (3)	0.07 (0.46)
Time off work (Days off work)	8.92 (30.71)	13 (16)	3.08 (11.55)	66 (24)	10.67 (34.25)
	n (%)		n (%)		n (%)
Investigations^c^ (MRI)	13 (4)		1 (1)		12 (4)
Investigations^c^ (X-rays)	26 (7)		7 (8)		19 (7)
Prescribed medication^c^	105 (29)		18 (21)		87 (31)
Over the counter^c^ medications	94 (26)		24 (28)		70 (25)

*NHS* National Health Service costs, *MRI* Magnetic resonance imaging

^a^_Physiotherapy treatment sessions received as part of the study_

^b^_Hospital based or private practice based consultations_

^c^_The number (%) of participants reporting usage within the investigations, prescribed medication and out-of-pocket and prescribed categories are reported instead of mean (SD) because of multiple usage, purchases and/or prescriptions_

^d^_Physiotherapy visits over and above those that were delivered as part of the ATLAS study_

**Table 3:** Mean (SD) costs per patient over 12 months

Cost (£)				
	ALL	Referred leg pain	Sciatica	Mean difference (95% CI)
ATLAS physiotherapy	43.09 (24.42)	36.94 (22.46)	44.94 (24.72)	
	*n* = 368	*n* = 85	*n* = 283	
Primary care				
GP consultations	72.07 (107.65)	50.40 (88.35)	78.57 (112.12)	28.17 (2.84 to 49.48)
Practice nurse consultations	1.25 (5.44)	2.01 (7.26)	1.02 (4.74)	−0.99 (−2.74 to 0.39)
Consultations with other professionals	5.86 (16.32)	5.18 (15.79)	6.06 (16.49)	0.88 (−3.09 to 4.76)
Prescriptions	1.02 (1.65)	0.65 (1.15)	1.13 (1.76)	0.33 (−3.19 to 3.20)
Hospital-based care				
NHS professional visits	59.08 (169.59)	51.64 (206.40)	61.32 (157.22)	9.68 (−40.87 to 51.47)
Private professional visits	33.61 (177.62)	59.61 (274.68)	25.80 (135.34)	−33.81 (−98.19 to 19.68)
Investigations (MRI, X-rays)	35.97 (93.57)	34.03 (108.76)	36.55 (88.71)	2.52 (−29.54 to 23.75)
Other healthcare professionals				
Additional NHS physiotherapy	109.28 (172.03)	93.69 (147.62)	113.96 (178.67)	20.27 (−20.69 to 55.45)
Private physiotherapy	19.37 (96.43)	20.71 (121.94)	18.97 (87.58)	−1.74 (−31.85 to 19.05)
NHS ‘other’	1.76 (20.08)	3.2 (29.50)	1.32 (16.26)	−1.87 (−10.67 to 2.62)
Private ‘other’	0.99 (12.83)	0	1.29 (14.62)	1.29 (0.15 to 3.75)
Over the counter purchases	10.51 (24.29)	11.34 (30.83)	10.26 (22.02)	−1.08 (−9.09 to 5.25)
Work-related: Time-off work associated with condition	728.14 (2635.13)	216.05 (857.31)	881.94 (2952.15)	665.89 (349.61 to 1120.31)
Imputed Analysis		*n* = 157	*n* = 452	
NHS costs	296.56 (326.75)	249.70 (301.58)	312.85 (333.83)	
mean difference (95% CI)		63.14 (8.70 to 117.35)		
Societal costs	1106.13 (2328.54)	700.82 (1006.76)	1246.92 (2623.35)	
mean difference (95% CI)		546.11 (274.16 to 844.01)		

*NHS* National Health Service costs, *MRI* Magnetic resonance imaging, *C0049* Confidence interval

The mean (SD) total societal cost for all patients in the cohort was £1106.14 (2328.54) (Table 3). The largest proportion (65%) of costs incurred by society was from indirect costs (lost productivity) due to the symptoms of low back and leg pain, with overall total costs for the whole cohort amounting to £728.14 (2635.13). Only 26% of the societal cost was incurred by the NHS.

Over the period of 12 months, there were observed differences in uptake of primary care and hospital visits and visits to ‘other’ professionals, and societal costs between the two groups with the sciatica group being associated with more GP visits, investigations, prescribed medication and visits to hospital consultants and other health professionals. The mean NHS costs per patient were £313 for the sciatica group compared with £250 for referred leg pain group. During the 12- month follow-up, the mean number of days off work was higher in the sciatica group (10.7 days) than in the referred leg pain group (3.1 days). This translated into higher productivity costs in the sciatica group (£882) compared to the referred leg pain group (£216), this difference was statistically significant. Table 3 shows the disaggregated mean (SD) NHS and societal costs per patient for each group and total cost estimates for the imputed data analysis.

### Impact of patient characteristics on cost and quality of life

Tables 4 to 5 show regression coefficients (95% CIs) for each of the potential predictors of cost and quality of life, representing the additional change associated with one-unit changes in each of these factors. From an NHS perspective, the final model included only three of the pre-selected variables (*p* < 0.25), none of which had a statistically significant impact on healthcare costs (Table 4). However, it can be observed from the societal costs final model including five of the pre-selected variables (Table 4), that the care pathway in the ATLAS study which was defined as a course of physiotherapy of 3 or more sessions, and better general health scores, were significantly associated with reduced costs (*p* = 0.024 and *p* = 0.063 respectively). The quality of life final regression model included all pre-selected variables, seven of which were significantly associated with poor quality of life (*p* < 0.05), these were: poorer self-reported general health, higher low back or leg pain intensity, higher depression score, higher disability score, patient’s belief/perception that their symptoms will be long-lasting, gender, and the ATLAS care pathway defined as a referral to spinal specialist services (Table 5).

**Table 4:** Factors associated with costs at 12 months, based on a generalised linear model for the whole group

	Coefficient (SE) *n* = 608
NHS Cost	
Constant	5.95 (0.163) ^**^
General Health	
SF-1 general health	−0.051 (0.055)
RMDQ	−0.000 (0.009)
Psychological measures and perceptions	
HADs depression	−0.016 (0.014)
AIC: 13.39 BIC: − 3303.19	
Societal Costs	
Constant	8.12 (0.403) ^**^
General Health	
SF-1 general health	−0.173 (0.099)^*^
RMDQ	−0.009 (0.018)
Psychological measures and perceptions	
HADs depression	−0.013 (0.026)
Personal characteristics	
Age^a^	−0.005 (0.006)
Comorbidities^b^	−0.050 (0.170)
Care pathways-unadjusted (0–2 physiotherapy sessions)	
3 or more physiotherapy sessions	−0.307 (0.160)^*^
Referrals to spinal specialist services	−0.174 (0.256)
AIC:16.00 BIC:-2441.59	

*NHS* National Health Service, *RMDQ* Roland Morris Disability Questionnaire, *SE* Standard Error, *HADs* Hospital and Anxiety Depression scale

^**^p < 0.05,^*^p < 0.1

^a^Sex is measured as 1 Female 0 Male

^b^Comorbidities included none, one, 2 or more chest problems, heart problems, raised BP, diabetes, circulation problems in leg(s)

**Table 5:** Generalised linear model with total QALYs at 12 months for the whole group

	Coefficient (SE) *n* = 557
Constant	0.577 (0.140)^**^
General Health	
SF-1 general health	−0.079 (0.019)^***^
RMDQ	−0.008 (0.004)^**^
Pain variables	
Duration of current episode of leg pain (< 6 weeks)	
6–12 weeks	−0.014 (0.041)
Over 3 months	−0.049 (0.037)
Pain intensity– highest of leg or back pain	−0.066 (0.012)^***^
Psychological measures and perceptions	
Illness perception:	
Identity^d^	−0.006 (0.013)
Timeline acute^a^ (Strongly disagree/disagree/neither)	
Agree/strongly agree	−0.079 (0.032)^**^
HADs depression	−0.022 (0.004)^***^
Personal characteristics	
Age	−0.002 (0.001)
Sex (Female)^b^	0.067 (0.032)^**^
BMI	−0.003 (0.002)
Comorbidities^c^	−0.038 (0.036)
Care pathways-unadjusted (0–2 physiotherapy sessions)	
3 or more physiotherapy sessions	0.004 (0.032)
Referrals to specialist spinal services	−0.119 (0.051)^**^
AIC: 0.99 BIC: − 3333.46	

*RMDQ* Roland Morris Disability Questionnaire, *SE* Standard Error, *HADs* Hospital and Anxiety Depression scale

^***^p < 0.01, ^**^p < 0.05

^a^Timeline; illness/condition duration: ‘my back and / or leg problem will last for a long time’). Timeline is measured on a Likert scale; strongly disagree - Disagree - Neither agree or disagree - Agree - Strongly agree. For the purposes of the analysis it was dichotomised ((agree (agree, strongly agree) versus disagree (strongly disagree, disagree, neither agree nor disagree))

^b^Sex is measured as 1 Female 0 Male

^c^Comorbidities included none, one, 2 or more chest problems, heart problems, raised BP, diabetes, circulation problems in leg(s)

^d^Identity; Symptom attribution to the condition from a list of 7 potential symptoms: back pain, leg pain, unable to sit comfortably, fatigue, stiff joints, sleep difficulties, loss of strength. The score is the sum of symptoms experienced The list of the 7 potential symptoms was chosen by the research team

### Sensitivity analysis

The results of the model analyses for costs and quality of life in the sciatica subgroup analyses were in line with base-case (whole cohort) findings. The exception in the analysis of quality of life was that the disability and care pathway variables were no longer significant for the sciatica group (See Additional files 4 and 5). Similar results were also observed in the regression analyses based on the complete-case analysis (See Additional files 6 and 7).

## Discussion

In this paper we report the total costs and quality of life outcomes of primary care patients seeking care for symptoms of back-related leg pain including sciatica, and we describe the factors associated with costs and quality of life outcomes in this patient group. To our knowledge, this the first study to provide information on costs (NHS and broader societal costs) and quality of life health outcomes, and on factors associated with these, in this population.

Our results from the overall cohort analyses demonstrate that healthcare utilisation from the NHS perspective is highest for health professional consultations such as physiotherapy and GPs. From a societal perspective, costs were highest for work-related productivity loss (for those in work). Similar findings were observed in the analyses for the sciatica and referred leg pain groups. An increase in the number of physiotherapy sessions (as part of the ATLAS study) and higher self-reported general health scores were significantly associated with reduced resource utilisation and costs at 12 months (approximately £1.5 and £1.2 respectively). The baseline factors associated with improvement of quality of life in this cohort were: higher scores of self-rated general health, lower pain intensity, lower depression scores, and lower disability scores. However, the disability score was not a significant predictor of quality of life in the sciatica group. A further finding was a higher number of days off work in the sciatica group within the 12 months follow-up, resulting in higher societal costs.

LBP national and international guidelines have recommended early assessment, diagnosis and management of back-related leg pain in patients presenting with back-related problems, with the view to prioritise timely and appropriate management [12, 38]. However, information on how costs and health related quality of life outcomes vary between patients with sciatica and referred leg pain remains limited, and patterns of how costs and outcomes change over time, have not been explored. No previous studies have assessed the impact of patient demographic and clinical characteristics on the costs and quality of life outcomes for this primary care population.

We did not find any significant association between costs and age, gender, body mass Index, comorbidities, disability, pain intensity, and depression in our sample. This contrasts with more general literature which suggests that costs of musculoskeletal conditions are often associated with demographic factors such as age, and disease specific factors such as depression and disability [39–43]. Factors significantly associated with quality of life outcomes in this cohort included general health, disability, pain intensity, and depression.

The strengths of our study lie in the analysis approaches used, presentation of disaggregated results and comprehensive assessment of factors associated with costs and quality of life in patients consulting with symptoms of low back-related leg pain including sciatica. The analyses are also performed from a broader societal perspective, and therefore report important work-related outcomes that are particularly relevant for this patient group, as most are of a working age. The analysis considers a comprehensive set of potential prognostic factors influencing cost and quality of life, underpinned by previous research and clinical experience. However, there are limitations to our analysis. Resource use information was collected using self-reported data from study participants at 4 and 12 months questionnaires. A limitation of using self-report data only, is that it is subject to recall and information bias (e.g patients not able to distinguish between LBP-related resource use and others) and therefore respondents could potentially over-report or under-report resource utilisation, particularly over longer periods of recall [44]. Nevertheless self-reported data provide an efficient means of obtaining economic evaluation data in the absence of routine data sources and has been widely used in similar studies [45–47]. Response rates for the main cost (60% at 12 months) and EQ-5D 3 L outcome data (97% at baseline, 64% at 4 months and 68% at 12 months) were low raising some concerns about the validity of the findings. However, appropriate and robust multiple imputation approaches were used to address potential biases resulting from incomplete data. In addition, data for the ATLAS study clinic sessions (which was significantly associated with total costs) was observed from routine physiotherapy databases, and could have influenced the overall results of the model. Lastly, although we evaluated costs from a societal perspective, we were not able to include all components of the indirect costs. Our study therefore presents conservative estimates of the total societal costs since presenteeism related costs were not included in the cost analysis. These costs have been shown to be greater than absenteeism in some cases [48].

The analysis reported here presents important information on costs for treating patients with back-related leg pain including sciatica. Within the ATLAS cohort, patients with sciatica pain and referred leg pain appeared to be similar in terms of quality-of-life outcomes although patients in the sciatica group had a higher proportion of consultation visits, medication and time-off work. The productivity costs incurred by individuals in this cohort as a result of back-related leg pain were substantial. Therefore it is important that the cost-effectiveness of interventions used for patients with low back-related leg pain and sciatica, are investigated from a perspective of both, the health provider and society.

The regression analyses showed that patient self-rated general health and the number of physiotherapy sessions received (as part of the ATLAS study) were important predictors of resource utilisation and costs, possibly indicating that initial comprehensive treatment seems to reduce future need for health care use and costs. However, the study’s observational design precludes any causal inferences. Baseline low levels of disability, pain intensity and depression were associated with changes in quality of life as measured by the EQ-5D-3 L. The generalizability of the findings in this research is supported by the inclusion of eligible patients with any level of intensity and duration of pain, as this broadens the population beyond patients presenting with only the most severe symptoms.

## Conclusion

In conclusion, in 1 year, resource utilisation for primary care patients with low back-related leg pain and sciatica showed that health care and societal costs were highest for visits to physiotherapists and GPs and in relation to work-related productivity loss. Health-related quality of life was low at baseline for the overall cohort and for both the sciatica and referred leg pain groups, but improved across all time points. The type of care received and patient self-reported general health were important variables for predicting future costs. Similarly to other musculoskeletal conditions, quality of life was significantly influenced by characteristics such as levels of disability, depression and pain intensity. Our study contributes to understanding the economics of low back-related leg pain and sciatica care and has important implications for health care policy resource allocation in this patient group.

## Additional files

Additional file 1: List of the preselected sets of variables used in the analysis. This additional file includes variables that have been preselected and included in the models. (DOCX 19 kb)

Additional file 2: Health care resource use unit costs. This additional file includes details of unit costs for variables used in the cost analyses. (DOCX 14 kb)

Additional file 3: Baseline characteristics for the whole group and for the sciatica and referred leg pain subgroups. This additional file provides a summary of baseline characteristics for the whole group. (DOCX 15 kb)

Additional file 4: Sensitivity analysis: Generalised linear regression model with NHS costs at 12 months (assuming a GAMMA Variance function, a log Link) for the sciatica group. This additional file reports model results of the sensitivity analysis of NHS costs for the sciatica group. (DOCX 15 kb)

Additional file 5: Generalised linear regression model with total QALYs at 12 months for the sciatica group. This additional file reports model results of the sensitivity analysis of total QALYs for the sciatica group. (DOCX 15 kb)

Additional file 6: Generalised linear regression model with NHS and Societal costs at 12 months complete-case. This additional file reports model results of the sensitivity analysis of NHS and societal costs for the complete-case analysis. (DOCX 16 kb)

Additional file 7: Generalised linear regression model with total QALYs at 12 months (complete-case). This additional file reports model results of the sensitivity analysis of total QALYs for the complete-case. (DOCX 17 kb)

## Data Availability

The datasets used and/or analysed during the ATLAS study are available from the corresponding author on reasonable request. Applications for access to anonymised data from our research databases are reviewed by the Centre’s Data Custodian and Academic Proposal (DCAP) Committee, and a decision regarding access to the data is made subject to the National Research Ethics Service ethical approval first provided for the study. Consideration to release data will also be undertaken in conjunction with journal publication and/ or funder restrictions that may apply. Further information on our data sharing procedures can be found on the Centre’s website (https://www.keele.ac.uk/pchs/datasharing/ /) or by emailing the Centre’s data manager (primarycare.datasharing@keele.ac.uk).

## Abbreviations

ATLAS: Assessment and treatment of leg pain associated with the spine
BNF: British National Formulary
GLM: Generalised linear models
GP: General Practitioner
LBP: Low back pain
MRI: Magnetic resonance imaging
NHS: National Health Service
QALY: Quality-adjusted life year
