# Supplementary material for: Factors associated with costs and health outcomes in patients with Back and leg pain in primary care: a prospective cohort analysis
Source: BMC Health Serv Res. 2019 Jun 21;19:406. doi: 10.1186/s12913-019-4257-0 (PMC6588896; doi:10.1186/s12913-019-4257-0)
Supplement: Supplementary file 1 — List of the preselected sets of variables used in the analysis. This additional file includes variables that have been preselected and included in the models. (DOCX 19 kb) [file 12913_2019_4257_MOESM1_ESM.docx]

Additional file 1: List of the preselected sets of variables used in the analysis.

| **Variable Set** | **Domain** | **Measure** |
| --- | --- | --- |
| **1** | Duration of pain | Current episode of leg pain: Less than 6 weeks, Between 6 to 12 weeks, Over 3 months (Dunn et al 2010). |
| **2** | Pain intensity | Taking the highest of either back or leg pain using the mean of three 0 to 10 numerical rating scales for ‘least’, ‘usual’ and ‘current’ pain over the previous 2 weeks (Dunn et al 2010) |
| **3** | Disability | Measured by the Roland Morris Disability Questionnaire (RMDQ) (Roland and Morris 1983) |
| **4** | Psychological perceptions | Pain self-efficacy: Measured with the Pain Self-Efficacy Questionnaire (PSEQ); with scores from 0 to 60; higher scores reflect stronger self-efficacy beliefs (Nicholas 2007)  Identity; Symptom attribution and causes of the condition (Moss-Morris et al 2002) from a list of 7 potential symptoms: back pain, leg pain, unable to sit comfortably, fatigue, stiff joints, sleep difficulties, loss of strength. The score is the sum of symptoms experienced. The list of the 7 potential symptoms was chosen by the research team.  Timeline; illness/condition duration: ‘ My back and / or leg problem will last for a long time’)**^a^**  Depression; Measured using the Hospital Anxiety and Depression scale (HADs); with scores from 0 to 21, higher scores indicate higher levels of depressive symptoms (Zigmond and Snaith 1981) |
| **5** | General Health | Respondent general health: Measured using the SF-1 general health question ranging from excellent to poor. (Ware 2000) |
| **6** | Quality of life | Respondent self-reported quality of life: Measured by the EQ-5D-3L questionnaire ( EuroQol Group 1990) |
| **7** | Socio-demographics | Age, gender, Body Mass Index, Comorbidities |
| **8** | Treatment | Care pathways: 0-2 physiotherapy sessions, 3 or more physiotherapy sessions, Referrals to specialist spinal services |

Timeline is measured on a Likert scale; Strongly disagree - Disagree - Neither agree or disagree - Agree - Strongly agree. For the purposes of the analysis it was dichotomised ((agree *(agree, strongly agree)* versus disagree *(strongly disagree, disagree, neither agree or disagree)*).

1. Dunn KM, Jordan KP, Croft PR. Recall of medication use, self-care activities and pain intensity: a comparison of daily diaries and self-report questionnaires among low back pain patients. *Primary Health Care Research & Development* 2010; 11:93-102.
2. Roland MO, Morris RW. A study of the natural history of back pain. Part 1: Development of a reliable and sensitive measure of disability in low back pain. *Spine* 1983; 8: 141-144
3. Nicholas MK. The pain self-efficacy questionnaire: Taking pain into account. *Eur J Pain* 2007; 11:153-163.
4. Moss-Morris R, Weinman J, Petrie KJ, Horne R, Cameron LD, Buick D. The Revised Illness Perceptions Questionnaire (IPQ-R). *Psychology and Health* 2002; 17(1): 1-16.
5. Zigmond AS, Snaith RP. The Hospital Anxiety and Depression Scale. *Acta Psychiatr Scand* 1983; 67:361-370.
6. Ware JE. SF-36 Health Survey Update. Spine. 2000a; 25(24):3130–3139.
7. EuroQol Group. EuroQol--a new facility for the measurement of health-related quality of life. The EuroQol Group. *Health Policy* 1990; 16:199-208.
