# Supplementary material for: Factors associated with costs and health outcomes in patients with Back and leg pain in primary care: a prospective cohort analysis
Source: BMC Health Serv Res. 2019 Jun 21;19:406. doi: 10.1186/s12913-019-4257-0 (PMC6588896; doi:10.1186/s12913-019-4257-0)
Supplement: Supplementary file 2 — Health care resource use unit costs. This additional file includes details of unit costs for variables used in the cost analyses. (DOCX 14 kb) [file 12913_2019_4257_MOESM2_ESM.docx]

Additional file 2: Health care resource use unit costs.

| Health care resource | Unit cost (£) |
| --- | --- |
| Primary care^a^ |  |
| General Practitioner: surgery consultation | 34 |
| Practice Nurse: surgery consultation | 9 |
| Practice Nurse: home visit | 20 |
| Community physiotherapist per hour | 30 |
| Hospital care contacts^b^ |  |
| A&E consultant | 111 |
| Outpatient consultant | 119 |
| Other health care contacts^c,d^ | 44 |
| Diagnostic tests: X-ray | 34.9 |
| Diagnostic tests: CT scan | 98 |
| Diagnostic tests: MRI scan | 169 |
| Out-of-pocket treatments | Patient reported costs |
| Prescribed medication^d^ | Patient-specific^d^ |
| Periods of work absence | Patient-specific^e^ |

^a Curtis L Unit Cost of Health and Social Care 2013/2014 PSSRU,^

^b NHS Reference costs schedule 2013/2014,^

^c Hospital-based or private practice, e.g. physiotherapy, acupuncture, osteopathy etc.^

^d NHS Reference costs schedule 2013/2014,^

^Formulary. 55th ed. London: BMJ Books, 2014^

^e Annual survey of hours and earnings (ASHE). London: Office for National Statistics, 2014.^
