# Supplementary material for: Factors associated with costs and health outcomes in patients with Back and leg pain in primary care: a prospective cohort analysis
Source: BMC Health Serv Res. 2019 Jun 21;19:406. doi: 10.1186/s12913-019-4257-0 (PMC6588896; doi:10.1186/s12913-019-4257-0)
Supplement: Supplementary file 3 — Baseline characteristics for the whole group and for the sciatica and referred leg pain subgroups. This additional file provides a summary of baseline characteristics for the whole group. (DOCX 15 kb) [file 12913_2019_4257_MOESM3_ESM.docx]

Additional file 3: Baseline characteristics for the whole group and for the sciatica and referred leg pain subgroups

|  | ALL | Referred leg pain | Sciatica |  |
| --- | --- | --- | --- | --- |
| Sociodemographic (Denominator*) | n = 609 | n = 157 | n = 452 | |
| Age (years), (609) Mean (SD) | 50.2 (13.9) | 49.4 (13.7) | 504.4 (14.0) | |
| Gender (609), Female | 381 (62.6) | 105 (66.9) | 276 (61.1) | |
| Current smoker (609) | 194 (31.9) | 43 (27.4) | 151 (33.4) | |
| Comorbidities^ⱡ^  (609) |  |  |  | |
| None | 371 (60.9) | 94 (59.9) | 277 (61.3) | |
| One other health problem | 158 (25.9) | 36 (22.9) | 122 (27.0) | |
| Two or more other health problems | 80 (13.1) | 27 (17.2) | 53 (11.7) | |
| BMI, mean (SD) (609) | 29.9 (7.0) | 30.0 (8.7) | 29.9 (6.3) | |
| HAD’s depression subscale, mean (SD) (609) | 6.4 (4.0) | 6.4 (4.0) | 6.3 (4.0) | |
| Leg pain intensity (608), mean (SD) | 5.2 (2.4) | 4.1 (2.3) | 5.6 (2.3) | |
| Back pain intensity (609), mean (SD) | 5.6 (2.2) | 5.4 (2.1) | 5.6 (2.2) | |
| RMDQ disability score (609) (0-23), mean (SD) | 12.7 (5.7) | 12.0 (5.7) | 12.9 (5.7) | |
| Illness perception (609) – Identity score, mean (SD) | 5.9 (1.3) | 5.9 (1.2) | 5.9 (1.3) | |
| Illness perception – Time-line (back/leg pain will last forever” [agree/strongly agree] (609) | 345 (56.7) | 96 (61.2) | 249 (55.1) | |
|  |  |  |  | |

BMI, body mass index; HADs, Hospital Anxiety and Depression scale; RMDQ, Roland-Morris Disability Questionnaire; SD, standard deviation

Note: All figures are frequencies and percentages (%), unless stated otherwise as mean and SD**.**

^ⱡ^ The health problems included chest problems, heart problems, raised blood pressure, diabetes, and circulation problems in the leg^.^

* The number of participants for each variable is shown in parentheses—the denominator varies for some participants due to missing data or not applicable case.
