# Supplementary material for: Factors associated with costs and health outcomes in patients with Back and leg pain in primary care: a prospective cohort analysis
Source: BMC Health Serv Res. 2019 Jun 21;19:406. doi: 10.1186/s12913-019-4257-0 (PMC6588896; doi:10.1186/s12913-019-4257-0)
Supplement: Supplementary file 4 — Sensitivity analysis: Generalised linear regression model with NHS costs at 12 months (assuming a GAMMA Variance function, a log Link) for the sciatica group. This additional file reports model results of the sensitivity analysis of NHS costs for the sciatica group. (DOCX 15 kb) [file 12913_2019_4257_MOESM4_ESM.docx]

Additional file 4: Sensitivity analysis: Generalised linear regression model with NHS costs at 12 months (assuming a GAMMA Variance function, a log Link) for the sciatica group

| Coefficient (SE) n = 451 | |
| --- | --- |
| **NHS Perspective** |  |
| Constant | 6.059 (0.179)** |
| General Health |  |
| SF-1 general health | -0.069 (0.061) |
| RMDQ | 0.000 (0.011) |
| Psychological measures and perceptions |  |
| HADs depression | -0.015 (0.015) |
| AIC: 13.50 BIC: -2313.62 |  |
| **Societal** |  |
| General Health |  |
| SF-1 general health | -0.158 (0.114) |
| RMDQ | -0.024 (0.021) |
| Psychological measures and perceptions |  |
| HADs depression | -0.009 (0.0301) |
| Personal characteristics |  |
| Age | -0.007 (0.007) |
| Comorbidities | -0.148 (0.195) |
| Care pathways-unadjusted (0-2 Physiotherapy sessions) |  |
| 3 or more physiotherapy sessions | -0.283 (0.184) |
| Referrals to spinal specialist services | -0.247 (0.281) |
| AIC:16.18 BIC: -1864.23 |  |
| ^NHS National Health Service; RMDQ Roland Morris Disability Questionnaire; SE Standard Error; HADs Hospital and Anxiety Depression scale; SE- Standard Error; ** p<0.05,* p<0.1^ | |
