# Supplementary material for: Factors associated with costs and health outcomes in patients with Back and leg pain in primary care: a prospective cohort analysis
Source: BMC Health Serv Res. 2019 Jun 21;19:406. doi: 10.1186/s12913-019-4257-0 (PMC6588896; doi:10.1186/s12913-019-4257-0)
Supplement: Supplementary file 5 — Generalised linear regression model with total QALYs at 12 months for the sciatica group. This additional file reports model results of the sensitivity analysis of total QALYs for the sciatica group. (DOCX 15 kb) [file 12913_2019_4257_MOESM5_ESM.docx]

Additional file 5: Generalised linear regression model with total QALYs at 12 months for the sciatica group

| Coefficient (SE) n = 414 | |
| --- | --- |
| Constant | 0.580 (0.172) |
| General Health |  |
| SF-1 general health | 0.100 (0.023)** |
| RMDQ | -0.005 (0.004) |
| Pain variables |  |
| Duration of current episode of leg pain (<6 weeks) |  |
| 6-12 weeks | -0.002 (0.049) |
| Over 3 months | -0.019 (0.045) |
| Pain intensity (highest of leg or back pain) | -0.069 (0.014)** |
| Psychological measures and perceptions |  |
| Illness perception: |  |
| Identity^*^ | -0.012 (0.016) |
| Timeline acute |  |
| Agree/strongly agree | -0.078 (0.039)** |
| HADs depression | -0.005 (0.005)** |
| Personal characteristics |  |
| Age | -0.003 (0.001) |
| Sex | 0.093 (0.038)** |
| BMI | -0.002 (0.003) |
| Comorbidities | -0.039 (0.043) |
| Care pathways-unadjusted (0-2 Physiotherapy sessions) |  |
| 3 or more physiotherapy sessions | 0.027 (0.039) |
| Referrals to spinal specialist services | -0.065 (0.061) |
| AIC:1.01 BIC:-2329.92 |  |
| ^*** ** p<0.05, p<0.1† SE – Standard Error, Timeline; illness/condition duration: ‘my back and / or leg problem will last for a long time’). Timeline is measured on a Likert scale; strongly disagree - Disagree - Neither agree or disagree - Agree - Strongly agree. For the purposes of the analysis it was dichotomised ((agree (agree, strongly agree) versus disagree (strongly disagree, disagree, neither agree nor disagree)).^‡ ^Identity; Symptom attribution to the condition (Moss-Morris et al 2002) from a list of 7 potential symptoms: back pain, leg pain, unable to sit comfortably, fatigue, stiff joints, sleep difficulties, loss of strength. The score is the sum of symptoms experienced. The list of the 7 potential symptoms was chosen by the research team^  ^RMDQ Roland Morris Disability Questionnaire; SE Standard Error; HADs Hospital and Anxiety Depression scale^ | |
