# Supplementary material for: Factors associated with costs and health outcomes in patients with Back and leg pain in primary care: a prospective cohort analysis
Source: BMC Health Serv Res. 2019 Jun 21;19:406. doi: 10.1186/s12913-019-4257-0 (PMC6588896; doi:10.1186/s12913-019-4257-0)
Supplement: Supplementary file 6 — Generalised linear regression model with NHS and Societal costs at 12 months complete-case. This additional file reports model results of the sensitivity analysis of NHS and societal costs for the complete-case analysis. (DOCX 16 kb) [file 12913_2019_4257_MOESM6_ESM.docx]

Additional file 6: Sensitivity analysis: Generalised linear regression model with NHS and Societal costs at 12 months complete-case.

| **NHS Cost** | | |  |
| --- | --- | --- | --- |
| Coefficient (SE) n = 462 | | |  |
| Constant | 6.08(0.181) ** | |  |
| SF-1 general health | -0.091(0.059) | |  |
| Duration of most recent episode of leg pain(Less than 6 weeks) |  | |  |
| 6-12 Weeks | -0.175(0.132) | |  |
| Over 3 months | -0.017(0.109) | |  |
| HADs depression | -0.0146(0.013) | |  |
| ^AIC: 13.34 BIC: -2380.37^ |  | |  |
| **Societal Costs** |  | |  |
| Coefficient (SE) n = 482 | | | |
| Constant | | 7.912(0.334) ** | |
| SF-1 general health | | -0.209(0.114)* | |
| RMDQ | | -0.001(0.022) | |
| Back/Leg problem will last for a long time (Strongly disagree/disagree/neither) | |  | |
| Agree or strongly agree | | -0.045(0.185) | |
| HADs depression | | -0.013(0.031) | |
| Care pathways-unadjusted (0-2 physiotherapy sessions) | |  | |
| 3 or more physiotherapy sessions | | -0.289(0.188) | |
| Referrals to spinal specialist services | | -0.305(0.294) | |
|  | |  | |
| ^AIC:15.95 BIC:-2064.69^ | |  | |
| ^** p<0.05,* p<0.1^ |  | |  |

^NHS National Health Service; RMDQ Roland Morris Disability Questionnaire; SE Standard Error; HADs Hospital and Anxiety Depression scale^
