# Supplementary material for: Factors associated with costs and health outcomes in patients with Back and leg pain in primary care: a prospective cohort analysis
Source: BMC Health Serv Res. 2019 Jun 21;19:406. doi: 10.1186/s12913-019-4257-0 (PMC6588896; doi:10.1186/s12913-019-4257-0)
Supplement: Supplementary file 7 — Generalised linear regression model with total QALYs at 12 months (complete-case). This additional file reports model results of the sensitivity analysis of total QALYs for the complete-case. (DOCX 17 kb) [file 12913_2019_4257_MOESM7_ESM.docx]

Additional file 7: Sensitivity analysis: Generalised linear model with total QALYs at 12 months for the whole group (Complete-case)

| Coefficient (SE) n = 260 | |
| --- | --- |
| Constant | 0.607 (0.258)** |
| SF-1 general health | -0.101( 0.035)** |
| RMDQ | -0.018 (0 .007)** |
| Pain variables |  |
| Duration of current episode of leg pain (<6 weeks) |  |
| 6-12 weeks | -0.079(0.069) |
| Over 3 months | -0.171(0.065)** |
| Pain intensity– highest of leg or back pain | -0.051(0.022)** |
| Psychological measures and perceptions |  |
| Illness perception: |  |
| Identity ^‡^ | 0.006(0.022) |
| Timeline acute^†^ (Strongly disagree/disagree/neither) |  |
| Agree/strongly agree | -0.049(0.056) |
| HADs depression | -0.027(0.009)** |
| Personal characteristics |  |
| Age | -0.000(0.002) |
| Sex (Female) | 0.014(0.056) |
| BMI | -0.002(0.005) |
| Comorbidities | -0.023(0.061) |
| Care pathways-unadjusted (0-2 physiotherapy sessions) |  |
| 3 or more physiotherapy sessions | 0.079(0.058) |
| Referrals to specialist spinal services | -0.105(0.093)** |
| ^AIC: 1.136 BIC: -1316.29^ |  |
| ^***p<0.01 ** p<0.05,* p<0.1^  ^† Timeline; illness/condition duration: ‘my back and / or leg problem will last for a long time’). Timeline is measured on a Likert scale; strongly disagree - Disagree - Neither agree or disagree - Agree - Strongly agree. For the purposes of the analysis it was dichotomised ((agree (agree, strongly agree) versus disagree (strongly disagree, disagree, neither agree nor disagree)).^  ^†† Sex is measured as 1 Female 0 Male^  ‡ ^Identity; Symptom attribution to the condition from a list of 7 potential symptoms: back pain, leg pain, unable to sit comfortably, fatigue, stiff joints, sleep difficulties, loss of strength. The score is the sum of symptoms experienced. The list of the 7 potential symptoms was chosen by the research team; RMDQ Roland Morris Disability Questionnaire; SE Standard Error; HADs Hospital and Anxiety Depression scale^ | |
|  |  |
